# Transcriptome reveals key microRNAs involved in fat deposition between different tail sheep breeds

**DOI:** 10.1371/journal.pone.0264804

**Published:** 2022-03-01

**Authors:** Xiaojuan Fei, Meilin Jin, Yuqin Wang, Taotao Li, Zengkui Lu, Zehu Yuan, Huihua Wang, Jian Lu, Kai Quan, Ran Di, Caihong Wei

**Affiliations:** 1 Institute of Animal Sciences, Chinese Academy of Agricultural Sciences, Beijing, China; 2 Henan University of Science and Technology, Luoyang, Henan, China; 3 Lanzhou Institute of Husbandry and Pharmaceutical Sciences, Chinese Academy of Agricultural Sciences, Lanzhou, Gansu, China; 4 Joint International Research Laboratory of Agriculture and Agri-Product Safety of Ministry of Education, Yangzhou University, Yangzhou, Jiangsu, China; 5 National Animal Husbandry Service, Beijing, China; 6 Henan University of Animal Husbandry and Economy, Zhengzhou, Henan, China; Northwest University, UNITED STATES

## Abstract

MicroRNA (miRNA) is a kind of noncoding RNA whose function involved in various biological processes in neuronal maturation and adipocyte cells, such as differentiation, proliferation, development, apoptosis, and metabolism. Herein, miRNA-Seq was used to identify miRNAs in the tail fat tissue of Hu sheep (short-fat-tailed) and Tibetan sheep (short-thin-tailed). In this study, 155 differentially expression miRNAs (DE miRNAs) were identified, including 78 up-regulated and 77 down-regulated. Among these DE miRNAs, 17 miRNAs were reported and related with lipid metabolism. MiRanda and RNAhybrid software were used to predict the target genes of DE miRNAs, obtaining the number of targeting relationships is 38553. Target genes were enriched by Gene Ontology (GO) and Kyoto Encyclopedia of Genes and Genomes (KEGG). 742 terms and 302 single pathways are enriched, including lipid metabolic process, response to lipid, cellular lipid catabolic process, lipid catabolic process, cellular lipid metabolic process, inositol lipid-mediated signaling, calcium channel activity, PI3K-Akt signaling pathway, MAPK signaling pathway, ECM-receptor interaction, AMPK signaling pathway, Wnt signaling pathway and TGF-beta signaling pathway. Notably, miR-379-5p was associated with tail fat deposition of sheep. Dual-Luciferase reporter assays showed miR-379-5p and *HOXC9* had targeted relationship. The result of RT-qPCR showed that the expression trend of miR-379-5p and *HOXC9* was opposite. miR-379-5p was down-regulated and highly expressed in tail adipose tissue of Tibetan sheep. *HOXC9* was highly expressed in adipose tissue of Hu sheep. These results could provide a meaningful theoretical basis for studying the molecular mechanisms of sheep tail adipogenesis.

## Introduction

Sheep is an important livestock used for meat, milk, wool, and fur. About 11000 years ago, sheep was domesticated [1]. With the development of domestication, sheep were divided into fat-tailed and thin-tailed sheep and study implied fat-tailed sheep evolved from thin—tailed sheep before 5000 years ago [2]. The tail type was determined by the degree and shape of fat deposition along the tail vertebrae. According to this standard, sheep can be divided into five types: short-thin-tailed sheep, long-thin-tailed sheep, short-fat-tailed sheep, long-fat-tailed sheep, and fat-buttock sheep [3]. Up to now, fat-tailed sheep account for approximately 25% of the world’s sheep population [4]. Although, tail fat deposition is a way to store energy for surviving in the harsh environment. But too much fat is not convenient for breeding. With the improvement of living standards, people’ eating habits have also changed to favor lean-meat with high protein, so sheep tail fat is becoming less and less popular among producers and consumers.

The formation of sheep tail type was regulated by multiple genes. Omics technique, an efficient and accurate method was used to study the mechanism of tail type trait. Wang *et al*. through genome-wide analysis reveals *PDGFD*, which related to angiogenesis, was significantly selected in Chinese indigenous sheep breeds of differential tail type [5]. Zhu *et al*. used ovine high-density 600K SNP arrays to detected genes associated with fat deposition, including *PPARA*, *RXRA*, *KLF11*, *ADD1*, *FASN*, *PPP1CA*, *PDGFA*, and *PEX6* [6]. Based on Fst and hapFLK approaches, identified *HOXA11*, *BMP2*, *PPP1CC*, *SP3*, *SP9*, *WDR92*, *PROKR1* and *ETAA1* may have important function in the formation of fat tail [7]. Among Chinese indigenous sheep breeds with extreme tail types, Altay sheep and Tibetan sheep, *WDR92*, *TBX12*, *WARS2*, *BMP2*, *VEGFA*, *PDGFD*, *HOXA10*, *ALX4*, and *ETAA1* whose function related to fat metabolism were identified association with sheep tail types [8]. About transcriptome, the expression profile of lncRNA and mRNA were described between various sheep breeds. Wang *et al*. analyzed the transcriptome information of tail adipose tissue between small-tailed F2 hybrid of wild Argali sheep and fat-tailed bash bay sheep to find *SCD*, *PHYH* and *CPAM*, which were related with tail fat and help understand molecular mechanism of fat tail [9]. Researches have investigated the first profile of lncRNA between Lori-Bakhtiari (fat-tailed) and Zel (thin-tailed) Iranian sheep [10]. In this study, miRNA-Seq was used to obtain the first comprehensive miRNAs expression profile between Hu sheep (short-fat-tailed) and Tibetan sheep (short-thin-tailed) in sheep tail fat. This study identified some miRNAs that may play an important role in fat metabolism. These data will provide a meaningful theoretical basis for studying the molecular mechanisms of miRNAs in sheep tail adipogenesis.

## Materials and methods

### Ethics statement and sample collection

All animal experiments were allowed by the Science Research Department of the Institute of Animal Sciences, Chinese Academy of Agriculture Sciences (IAS-CAAS). And ethical approval was given by the Animal Committee of the IAS-CAAS (No. IAS 2020–82). Samples of ovine tail fat were collected from three Hu sheep (short-fat-tailed sheep, Yongdeng, Gansu, China) and three Tibetan sheep (short-thin-tailed sheep, Yushu, Qinghai). All sheep were males and slaughtered at age 1.5. Collecting tail fat tissue of each sheep, immediately frozen in liquid nitrogen in RNase-free 1.5 mL freezing tubes, and store at -80°C for use.

### miRNA library preparation and sequencing

Total RNA was extracted by TRIzol (Invitrogen, CA, USA) following the manufacturer’s instruction. Using NanoDrop2000 spectrophotometer to quantify RNA purity at 260 and 280 nm (Thermo Fisher Scientific, MA, USA). Integrity of RNA and library was examined by Agilent 2100 Bioanalyzer (Agilent Technologies, CA, USA). Six libraries were constructed, named HZ1, HZ2, HZ3, ZZ1, ZZ2 and ZZ3. All libraries were sequenced using BGISEQ-500 technology [11]. All FASTQ sequencing files have been stored in Sequence Read Archive (accession numbers PRJ NA 777369).

### Sequencing analysis

The raw sequencing data are called raw tags. Following these steps: remove low quality tags; remove tags with 5 primer contaminants; remove tags without 3 primer contaminants; remove tags without insertion; remove tags with poly A; remove tags shorter than 18 nt to obtain clean tags. After filtering, the clean tags were mapped to the reference genome of Oar_v3.1 (http://www.ensembl.org/Ovis_aries/Location/Genome?db=core) and miRbase21.0 (http://www.mirbase.org) with Bowtie2 [12]. miRDeep2 was used to predict novel miRNA by exploring the secondary structure [13]. Known miRNA were described with “oar-miR-”. Novel miRNAs were described with “novel_mir”.

### miRNA expression analysis

miRNAs expression level is calculated by counting absolute numbers of molecules using unique molecular identifiers [14]. After obtaining the clean tags, we divided these libraries into two groups, including HZ (HZ1, HZ2, HZ3) and ZZ (ZZ1, ZZ2, ZZ3). Using DESeq2 to performed the differential expression analysis of miRNAs [13]. The corrected *P* ≤0.05 and |Log2Foldchange |> 1 as the default threshold to judge the significance of expression difference.

### Target genes prediction and functional analysis of DE miRNAs

Using miRanda [15] and RNAhybrid [16] to predict target genes of differently expression miRNAs. The DE miRNAs target genes were annotated by Gene ontology (GO) (http://www.geneontology.org/) including the cellular component, biological process, and molecular function. KEGG biological pathways database (http://www.genome.jp) was used to enrich target genes. The *P* value was corrected using the Bonferroni method and *P* ≤ 0.05 was taken as significantly enriched terms [17].

### RT-qPCR

DE miRNAs were selected randomly and RT-qPCR was used to verify the accuracy of the sequencing data. Using Stem-loop method to synthesize cDNA from miRNAs and miRNA Design V1.01 and Primer 5.0 were used to design primers. miRNA 1st Strand cDNA Synthesis Kit and miRNA Universal SYBR qPCR Master Mix were (Vazyme, Nanjing, China) used. 5s was used to be housekeeping gene. HiScript III 1st Strand cDNA Synthesis Kit (+gDNA wiper) and ChamQ Universal SYBR qPCR Master Mix (Vazyme, Nanjing, China) were used to detected the expression of HOXC9. β-actin was used to be housekeeping gene. All primers sequences are listed in S1 Table. And the relative expression level of mRNA and miRNA were calculated using the 2^−ΔΔCt^ method.

### Dual-Luciferase reporter assays

To verify the target relationship between *HOXC9* and miR-379-5p. The wild-type 3’UTR of the *HOXC9* mRNA was amplified between the *Xho* I and *Not* I restriction enzyme cutting sites. The primers used in plasmid construction are designed by SnapGene and showed in S1 Table. The fragments were inserted into psiCHECK2 vector (Promega, WI, USA) and named psiCHECK2-*HOXC9*-3′UTR-WT. Site‐Directed Mutagenesis Kit (Thermo Fisher Scientific, MA, USA) was used to generate the mutant type of 3’UTR of *HOXC9* and named psiCHECK2-*HOXC9*-3′UTR-MT. There are four groups including psiCHECK2-*HOXC9*-3′UTR-WT with miR-379-5p mimics, psiCHECK2-*HOXC9*-3′UTR-MT with miR-379-5p mimics, psiCHECK2 pure vectors with negative control (NC) and psiCHECK2 pure vectors with miR-379-5p mimics for cell transfection. Lipofectamine 2000 Reagent (Thermo Fisher Scientific, MA, USA) was used to co-transfect into 293T cells. After incubation for 6 h, the culture medium was changed. After 48 hours of incubation, the relative luciferase activity in the cells was measured by Dual-Luciferase Reporter Assay System (Promega, Promega, WI, USA). Each treatment was performed 4 times for each group.

### Statistical analysis

The data of dual-luciferase reporter and RT-qPCR processed by T.TEST of Excel 2019. The results are presented as means ± standard deviation. Furthermore, *P* ≤ 0.05 was regarded as statistically significant, *P* ≤ 0.01 was highly significant, and *P* > 0.05 was not significant.

## Results

### Overview of miRNA sequencing

To identify differentially expressed miRNAs in HZ (HZ1, HZ2 and HZ3) and ZZ (ZZ1, ZZ2 and ZZ3), six libraries were constructed. The results of miRNA-Seq data of each library after quality controlled were shown in Table 1. The clean tag count of each sample ranged from 26 to 28 million and the Q20 of clean tag ranged 98.30 to 98.60%. About 76.37–89.01% of the clean reads were mapped to the sheep reference genome. Most of sequences were concentrated between 20 and 23nt. The number of sequences with 22nt was the most numerous, which is more than 30% (S2 Table). The further study of sequences, using miRDeep2 software to predict new miRNAs. In result, 130 miRNAs known 297 novel miRNAs and were found in HZ1, 131 miRNAs known 282 novel miRNAs and were found in HZ2, 134 miRNAs known 288 novel miRNAs and were found in HZ3, 140 miRNAs known 241 novel miRNAs and were found in ZZ1, 141 miRNAs known 231 novel miRNAs and were found in ZZ2, 139 miRNAs known 224 novel miRNAs and were found in ZZ3, respectively (S3 Table).

**Table 1:** Summary of raw tags after filtering and mapping to the reference genome of each library.

Sample name	Sequence type	Raw tag count	Clean tag count	Percentage of clean tag (%)	Q20 of clean tag (%)	Percentage of mapped tag (%)
HZ1	SE50	30,050,632	27,609,934	91.88	98.40	86.10
HZ2	SE50	29,091,769	28,219,709	97.00	98.40	88.93
HZ3	SE50	28,225,676	26,521,500	93.96	98.30	89.01
ZZ1	SE50	30,081,226	29,243,657	97.22	98.40	76.37
ZZ2	SE50	27,910,701	27,001,328	96.74	98.50	77.87
ZZ3	SE50	29,335,311	28,296,792	96.46	98.60	85.74

### Differentially expressed analysis of miRNA

**Fig 1:**
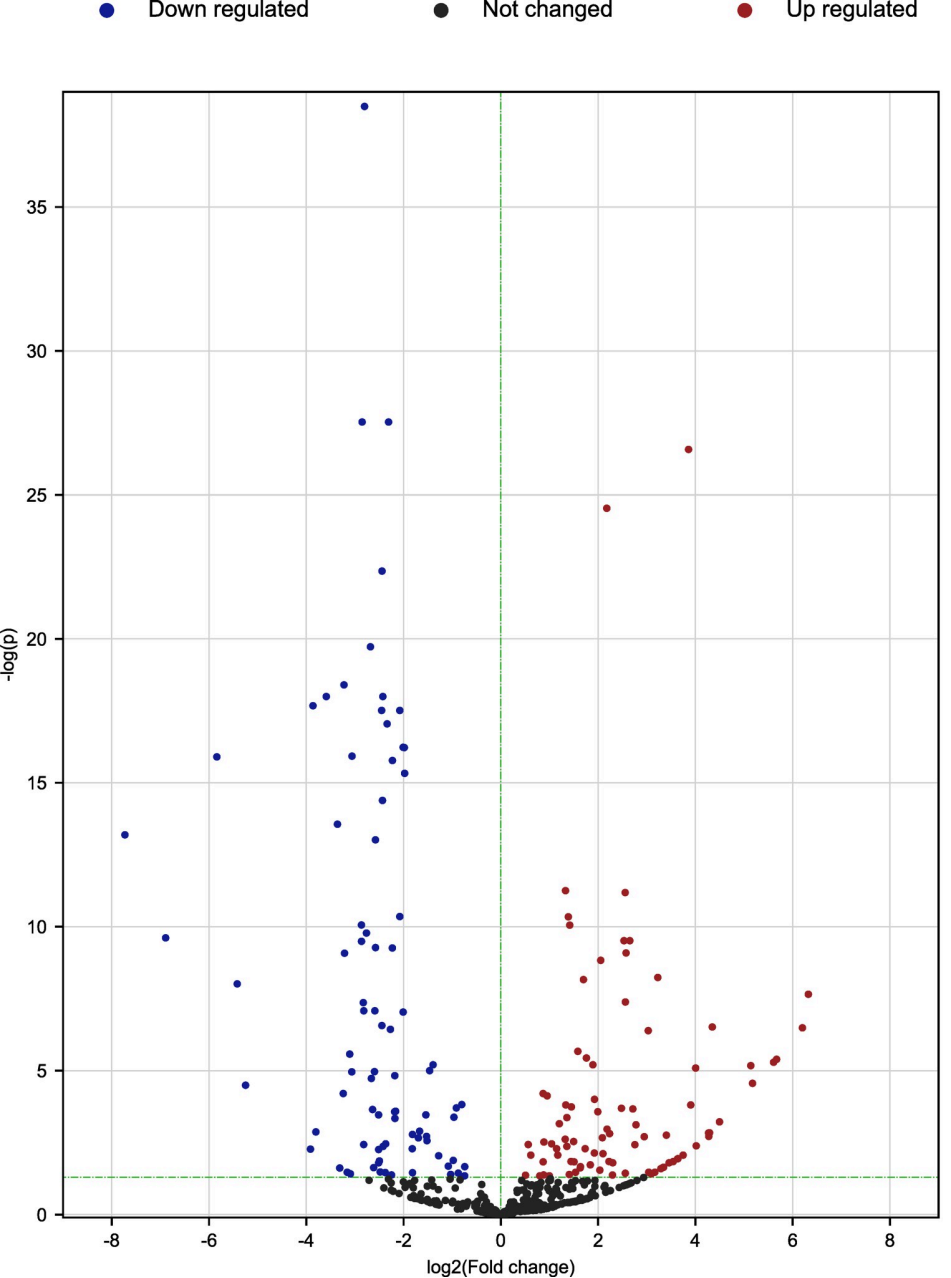
The volcano plots of differentially expressed genes between Hu sheep (HZ) and Tibetan sheep (ZZ) in fat tail. The x-axis shows the values of log2 (fold change), while the average expression values of -log10 (*p*) are displayed by the y-axis. The blue and red dots represent the significantly differentially expressed transcripts (*p* < 0.05) comparing the HZ and ZZ, with blue for downregulated genes and red for upregulated genes. The black dots indicate the transcripts with expression levels which are not statistically significant (*p* > 0.05) comparing HZ and ZZ.

In two comparisons, 147 known miRNAs and 389 novel miRNAs were identified (S4 Table). Based on the corrected *P* < 0.05, we detected 155 DE miRNAs in total, including 78 up-regulated and 77 down-regulated (Fig 1, S5 Table).

### DE miRNAs target prediction and functional analysis

**Fig 2:**
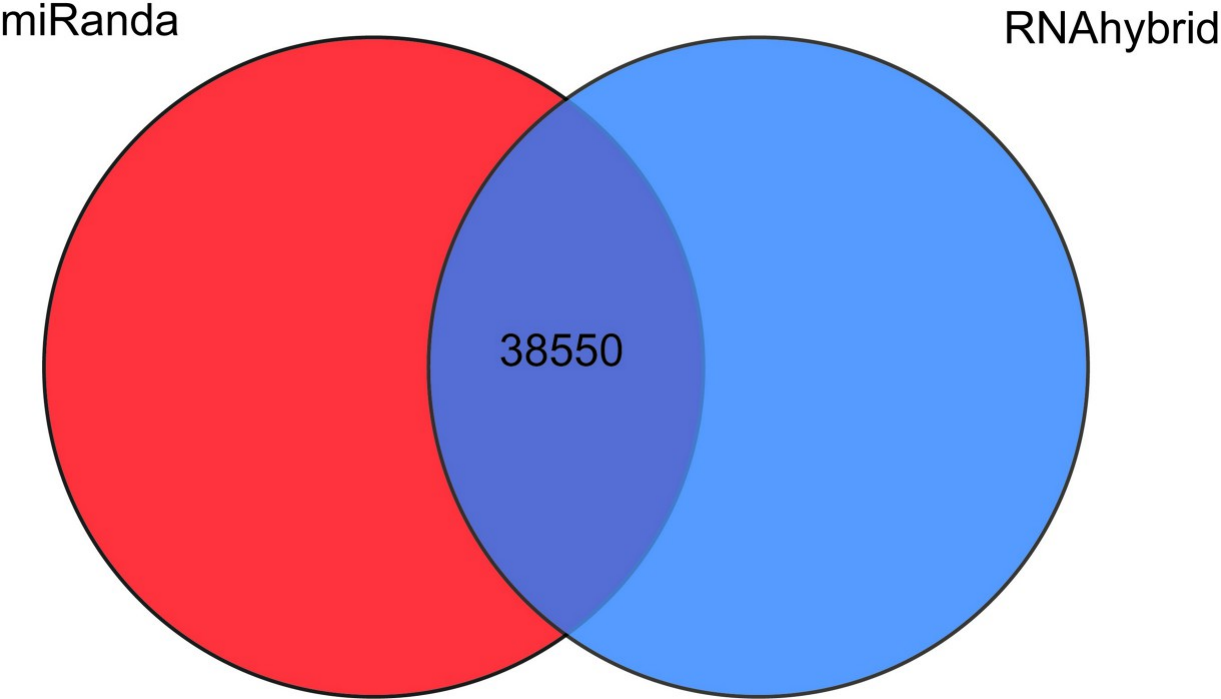
Venn diagram of targeting relationship of DE miRNAs. The number is the intersection of the relationship pairs predicted by RNAhybrid and miRanda.

**Fig 3:**
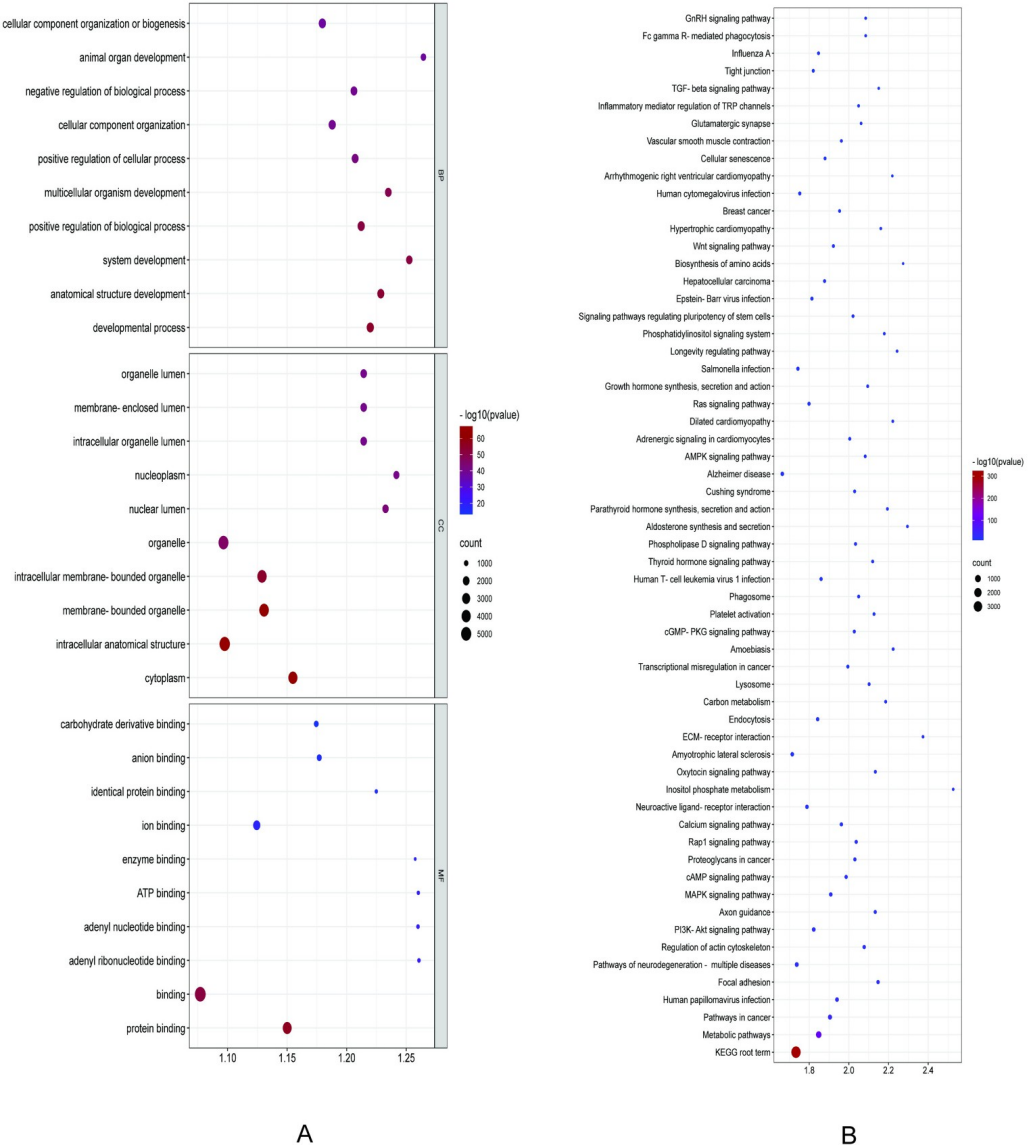
Target gene function enrichment of GO and KEGG. (A) Each top ten enrichment pathways for BP, CC, MF of target genes. The x-axis displays enrichment, and the y-axis represents the GO terms. The filled colored circles display each statistically significant GO term. The size of circles represents gene number. (B) Top sixty pathways of target genes. The x-axis displays rich factor of target genes, and the y-axis represents the KEGG pathway. The filled colored circles represent each statistically significant KEGG pathway. The filled colored circles display each statistically significant KEGG term. The size of circles represents gene number.

miRanda and RNAhybrid software were used to predict the target genes of DE miRNAs, resulting in number of predicted targeting relationships was 38553 in total (Fig 2, S6 Table). Go enrichment analysis showed that 557 terms were preferentially enriched in biological processes (BP), including lipid metabolic process, response to lipid, cellular lipid catabolic process, lipid catabolic process, cellular lipid metabolic process and inositol lipid-mediated signaling. 101 terms were preferentially enriched in cell components (CC). While 83 terms were significantly enriched in molecular functions (MF), including calcium channel activity (Fig 3A, S7 Table). Eventually, 302 signaling pathways significantly enriched, including PI3K-Akt signaling pathway, MAPK signaling pathway, ECM-receptor interaction, AMPK signaling pathway, Wnt signaling pathway, TGF-beta signaling pathway, and so on (Fig 3B, S8 Table). These results suggested that the target genes of DE miRNAs may be involved in the regulation of sheep tail type by participating in fat metabolic signaling pathways.

### Plasmid construction and identification

Selecting eight monoclonals randomly and using vector universal primers to identify the wild-type psiCHECK2 plasmid by polymerase chain reaction (PCR) and sequencing (S1 Fig, S9 Table). After sequence compared, the plasmid was constructed successfully. Primers of sequencing are shown in S1 Table. Eventually, site-directed mutation was used to obtain the mutant-type psiCHECK2 (S10 Table).

### Validation of miRNAs expression by RT-qPCR

**Fig 4:**
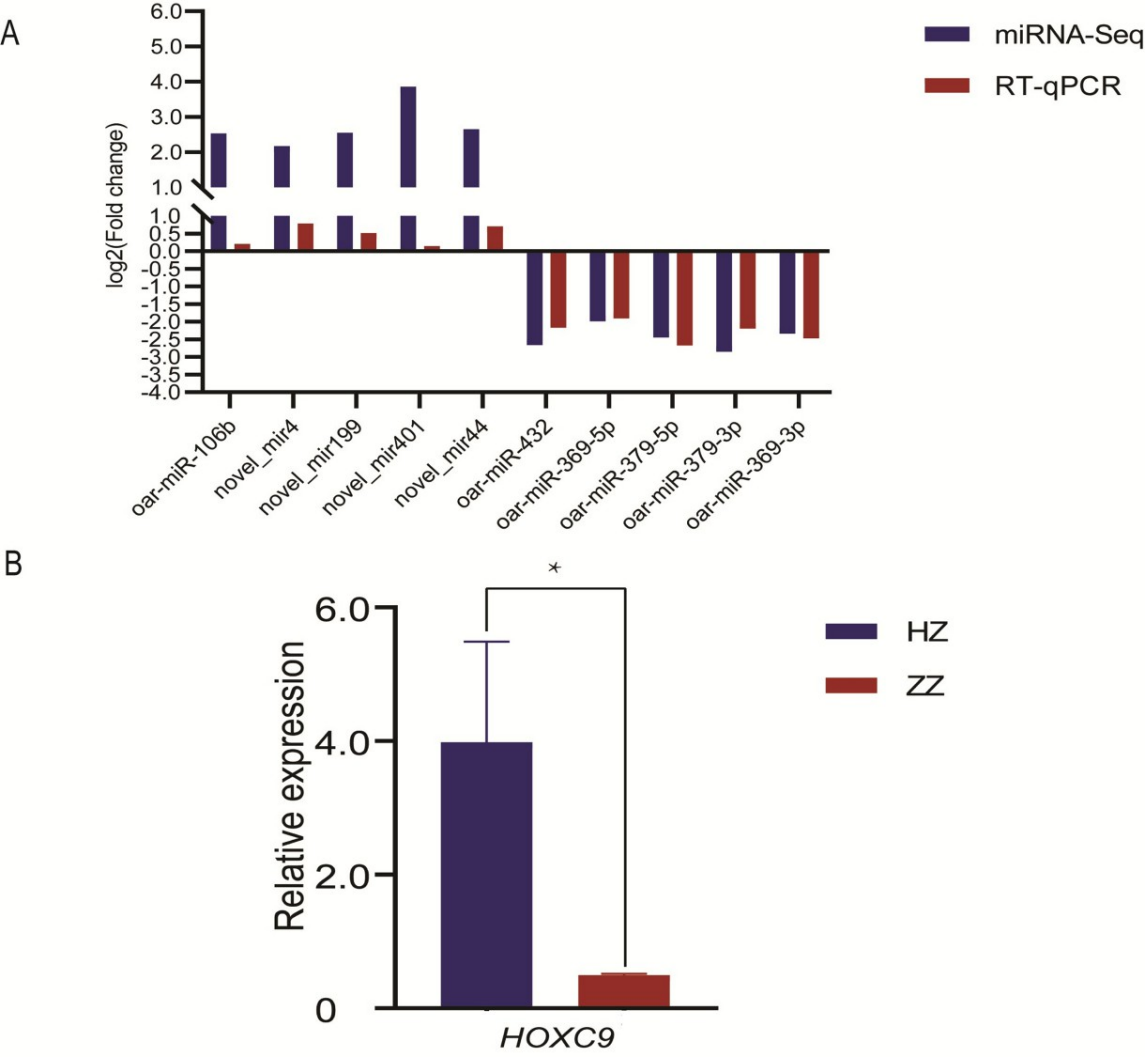
The result of RT-qPCR. (A) RNA-Seq and RT-qPCR results of ten selected differentially expressed miRNAs in HZ and ZZ. (B) RT-qPCR results of *HOXC9* in HZ and ZZ. * indicates significant difference (*P*<0.05).

The validation results for the ten miRNAs selected to substantiate the accuracy of sequencing are displayed in Fig 4A. oar-miR-106b, novel_mir4, novel_mir199, novel_mir401 and novel_mir44 were upregulated in tail fat of Hu sheep, and oar-miR-432, oar-miR-369-5p, oar-miR-379-5p, oar-miR-379-3p and oar-miR-369-3p were upregulated in tail fat of Tibetan sheep. The results indicate that there is a similar expression pattern of miRNAs generated from miRNA-Seq and RT-qPCR data.

### Validation of the target relationship between oar-miR-379-5p and *HOXC9*

**Fig 5:**
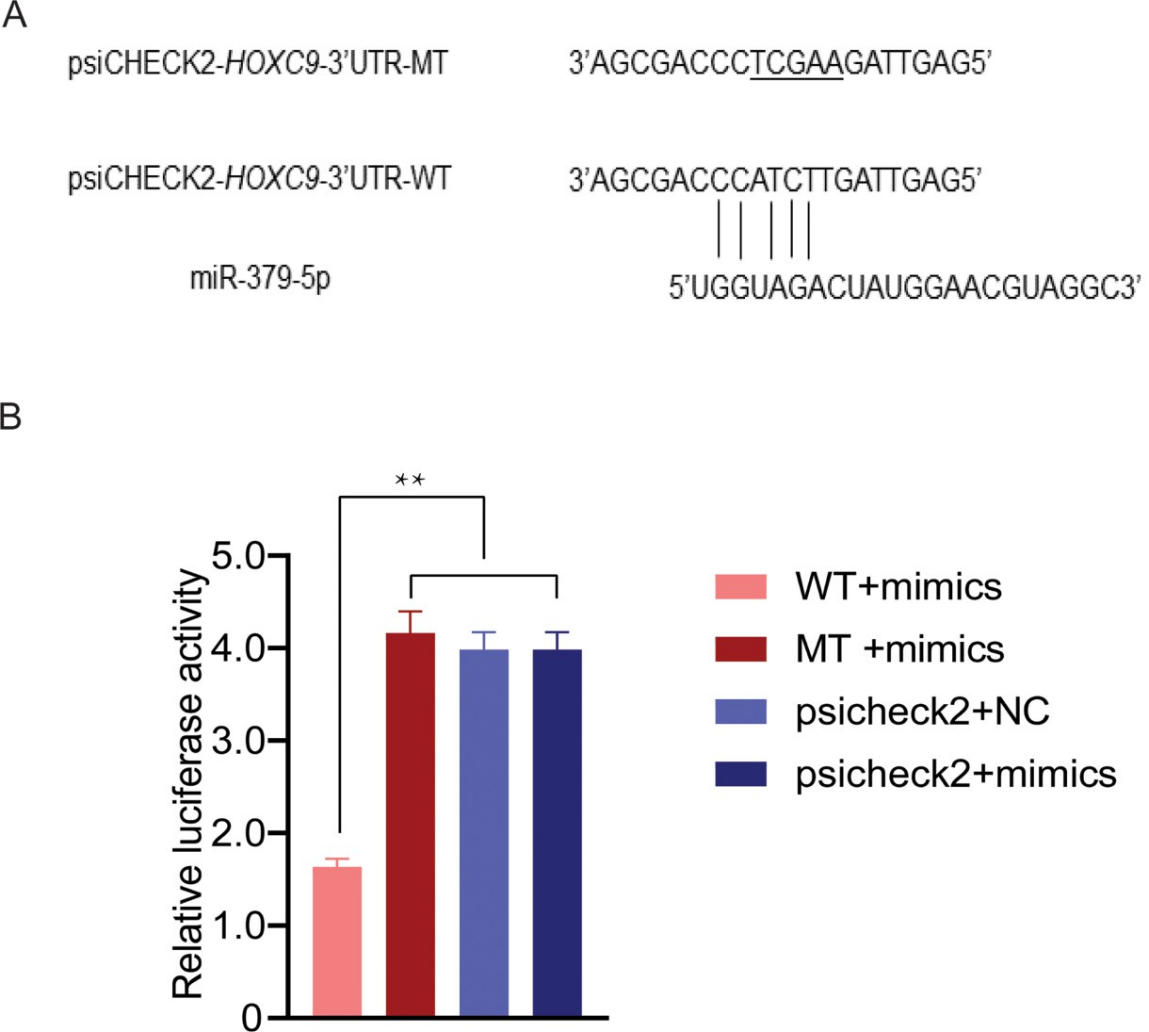
Result of the luciferase reporter assay. (A) Potential binding site between oar-miR-379-5p and *HOXC9*3’UTR. The underlined sequences represent the mutant sites. (B) WT display the psiCHECK2-*HOXC9*-3′UTR-WT. MT display psiCHECK2-*HOXC9*-3′UTR-MT. psiCHECK2 display psiCHECK2 pure vectors. Mimics display miR-379-5p mimics. ** indicates highly significant difference (*P*<0.01).

Dual-luciferase reporter assay indicated that oar-miR-379-5p significantly suppressed the luciferase activities for co-transfecting with wild types of *HOXC9* 3’UTR, while no effect on the mutant types of *HOXC9* 3’UTR or blank vectors (Fig 5B). These results initially confirmed the direct interactions between oar-miR-379-5p and *HOXC9*.

## Expression of *HOXC9*

RT-qPCR showed the expression of *HOXC9* in Hu sheep was significantly higher than that of Tibetan sheep (*P*<0.05) (Fig 4B).

## Discussion

MiRNA is endogenous single-stranded noncoding RNA whose length is approximately 22 nt. Adipose tissue is an endocrine organ which play an important role in regulation lipid metabolic in the organism [18]. To better understand the relationship between miRNAs and tail fat deposition, we identified and characterized the expression patterns of miRNAs in different tail sheep through high-throughput sequencing, as well as bioinformatics analysis. In our study, 17 miRNAs have been reported, which related with fatty metabolism. In human adipose tissue derived stromal cell, miR-369-5p [19] inhibited cell proliferation and adipocytes differentiation by targeting the regulation of *FABP4*, while miR-29b [20] promoted cell differentiation. Meanwhile, the expression level of miR-374a [21] could be used as an indicator to assess the occurrence of lipid metabolic diseases including diabetes. In human, miR-495-3p [22] was differentially expressed between fasting and postprandial states, and the target genes of DE miRNA were annotated in MAPK signal pathway and PI3K-Akt signaling pathway. Study found *AGT* contains a miR-31 polymorphic binding site, it can regulate distribution of body fat in men and women [23]. In mouse model, researchers found miR-379-5p [24], miR-103 [25], miR-194 [26] and miR-409-3p [27] may be a therapeutic target for lipid metabolism diseases. In adipocytes of mouse, miR-17-5p regulated *Tcf7l2* through Wnt signal pathway [28] to inhibit cell differentiation, but BPrdm16 was targeted regulation by miR-133 [29] can promote cell differentiation. Selecting the DE miRNAs between subcutaneous adipose-derived stem cells and omentum adipose-derived stem cells and predicting the target genes of DE miRNAs. Recent study found miRNAs had important function in lipid metabolism of livestock. MiR-25 repressed the expression of *PGC-1beta* to modulate triacylglycerol and lipid accumulation in goat mammary epithelial cells [30]. MiR-381 [31] targeted *KCTD15* to promoted triglyceride accumulation through in vitro culture bovine preadipocyte. Meanwhile, miR-432 [32] was highly expressed in back fat of cattle, which targeted *PRKAA1/2*, *PPARA* and *PPARG* to modulate lipid and fatty acid metabolism. Result of target gene function enrichment showed some pathways related fat metabolism, including PI3K-Akt signaling pathway, MAPK signaling pathway, ECM-receptor interaction, AMPK signaling pathway, Wnt signal pathway and TGF-beta signaling pathway. Studies showed these pathways had an important function in fat metabolism. PIK3 is a kind of intracellular lipid kinase, which phosphorylates phosphatidylinositol to produce an intracellular second messengers. These second messengers activate many signaling pathways to regulate biological processes in cells [33]. Current study reported that the PI3K signaling pathway participates in biological processes related to obesity. In mouse, insulin signaling via the PI3K/Akt axis account for the excess of lipids has to be properly stored in fat tissue [34]. PI3K/Akt pathway has different function between different adipocytes. In human adipocytes, PI3K/Akt pathway can promote the proliferation and differentiation [35], while, PI3K/Akt pathway can inhibit the proliferation and differentiation in 3T3-L1 [36]. In our study, PI3K/Akt pathway was enriched. This pathway may have vital function in adipose tissue of tail between Hu sheep and Tibetan sheep.

MAPK signaling pathway, including extra cellular signal-regulated kinase (Erk), p38, and c-Jun NH2-terminal kinase (JNK) [37]. The pathway played vital role in adipocyte proliferation and differentiation. A previous study regarding the adipocyte-specific transcription factor *PPARγ*, *C/EBPα*, *β*, and *δ*, can be phosphorylated by Erk1/2 to decrease its transcriptional activity and inhibit adipocyte differentiation [38]. In porcine adipocyte, miR-29a promotes adipocyte proliferation and inhibits adipocyte differentiation by targeting the regulation of *CTRP6* through the p38 MAPK pathway [39]. Coincidently, our study showed the target genes of DE miRNAs were annotated in MAPK pathway. MAPK pathway has different function in process of lipogenesis. At the individual level, p38 MAPK could inhibit adipogenesis differentiation by inhibiting the activity and expression of *C/EBPβ* and *PPARγ* during the whole process of lipogenesis in mice [40]. In human mesenchymal stem cells, researches verified that p38 the member of MAPK could promote clonal expansion during early lipogenesis. But in a later adipogenesis stage, p38 could inhibit the active of adipocyte-specific transcription factor [41].

The main constituents of Extracellular matrix (ECM)-receptor interaction signaling pathway in adipose tissue includes collagen (type I, IV, and VI), fibronectin (FN), laminin (LN1,8), hyaluronan, and proteoglycan [42]. Extracellular matrix components were predominantly released during the early and middle stages of 3T3-L1 differentiation, with a subsequent increase in the secretion of adipokines to promote lipid accumulation [42]. ECM-receptor interaction signaling pathway also has important function during differentiation of human mesenchymal stromal-cells into adipocytes [33]. These studies argued that the ECM-receptor interaction signaling pathway is essential for tissue architecture and has an important role in adipogenesis. Through RNA-Seq to identify genes in omental, subcutaneous and intramuscular fat of cattle, which was related fat metabolism. By functional analysis, ECM-receptor interaction signaling pathway was highly enriched. Between Zhuanghe dagu chicken and the Arbor Acres Broiler chicken, RNA-sequencing analysis of pectorales and crus showed some genes affect IMF deposition were significantly enriched in ECM-receptor interaction signaling pathway [43, 44]. Target genes of DE miRNAs were enriched in ECM receptor interaction signaling pathway. We speculate that ECM receptor interaction signaling pathway also plays an important role in fat metabolism of sheep tail.

AMPK pathway involves various activities, such as lipid metabolism [45], diseases [46] and growth [47]. Studies have shown that some drugs ameliorate lipid accumulation and inflammation in nonalcoholic fatty liver disease through the AMPK pathway, such as LB100 [48], Ursolic acid [49], Allyl isothiocyanate [50], Ginsenoside Rk3 [51] and Kangtaizhi Granule [52]. As reported, miR-122 promotes lipogenesis via inhibiting the AMPK pathway by targeting *SIRT1* in HepG2 and Huh-7 cells [53]. In our study, *SIRT1* was also enriched in this pathway. In summary, AMPK has an important function in lipid metabolism, which can be identified a potential pathway in fat metabolism of sheep.

In this study, the target genes of DE miRNA were enriched Wnt signal pathway. Investigations revealed the Wnt pathway had key function in regulating body mass, glucose metabolism, de-novo lipogenesis, low density lipoprotein clearance, vascular smooth muscle plasticity, liver fat and liver inflammation [54]. Wnt family genes were identified in mouse, recent study showed that Wnt signal pathway had important role in obesity and white fat browning process. Triazole-based can inhibit Wnt signal to improve glucose and lipid metabolism in diet-induced obese mice [55]. In 3T3-L1, TCF7L2 can improve triglyceride accumulation through Wnt signal pathway [56].

TGF-beta signaling pathway in adipocyte differentiation and lipid metabolism had important regulatory effects had an important function. In high-fat diet (HFD) induced nonalcoholic fatty liver disease (NAFLD) Sprague—Dawley rat models, Isoquercetin could treat NAFLD through TGF-beta signaling pathway. Researchers also found Isoquercetin can improve hepatic lipid accumulation and decrease inflammation and oxidative stress suppressing TGF-beta signaling pathway in co-culture cells model between primary hepatocytes and Kupffer cells induced by lipopolysaccharides/free fatty acids [57]. Addition, *BMPs* were related with fat formation. *BMP4* played an active function in fat biogenesis, which can facilitate beige fat biogenesis via regulating adipose tissue macrophages [58]. In diabetic mice and palmitate (PA)-induced insulin-resistant HepG2 and AML12 cells, *BMP7* can inhibit the active of *MAPKs* to regulate insulin resistance [59].

miRNAs can bind their target messenger RNAs (mRNAs) through either partial or perfect complementarity and promote their degradation or inhibit their translation to regular gene expression at transcriptional level [60]. Recent studies have shown that miRNAs can target mRNA to regulate adipocyte proliferation and differentiation in livestock. In porcine intramuscular preadipocytes, miR-125a-5p promoted proliferation and inhibited differentiation, which targeted *KLF13* and *ELOVL6* [61]. Meanwhile, miRNA-29b/29c targeted *CTRP6* to promoted proliferation and inhibited differentiation of porcine intramuscular preadipocytes [62]. In bovine preadipocyte, microRNA-1271 promotes differentiation by targeting activation transcription factor 3 [63]. miRNAs have also been found to play an important role in the fat cells of model animals. In 3T3-L1, miRNA-16e-5p promoted fat droplet accumulation and adipocyte differentiation [64]. In this study, miR-379-5p was differentially expressed in tail adipose tissue of Hu sheep and Tibetan sheep. Previous study found miR-379-5p were related with fat metabolism disease which can regulate LIN28/let‑7 to suppress diabetic nephropathy [26]. In this study, miR-379-5p was differentially expressed in tail adipose tissue of Hu sheep and Tibetan sheep. Using miRanda and RNAhybrid soft predicted *HOXC9* was the one of target genes of miR-379-5p. Researchers speculated *HOXC9* had important function in development of human obesity [65]. By comparison, *HOXC9* mRNA expression was significantly higher in abdominal subcutaneous and it significantly correlates with body fat mass. In both Siberian healthy miners living at extremely cold temperatures and healthy subjects living in thermoneutral conditions. Efremova A *et al*. used RT-qPCR to further confirmed the function of *HOXC9*, which was related to white adipocytes browning [66]. In GeneCards (https://www.genecards.org/), *HOXC9* was enriched in differentiation of white and brown adipocyte. In this study, we demonstrated the targeted relationship between oar-miR-379-5p and *HOXC9* in 293T. The result of RT-qPCR showed that the expression trend of miR-379-5p and *HOXC9* was opposite. miR-379-5p was highly expressed in tail adipose tissue of Tibetan sheep. *HOXC9* was highly expressed in adipose tissue of Hu sheep. The above results could verify miR-379-5p and *HOXC9* had targeted relationship, but they could not to illuminate the regulation mechanism of fat deposition in sheep tail. In future, the regulation mechanism of fat deposition in sheep tail between miR-379-5p and *HOXC9* need to verify.

## Conclusion

In this research, 155 DE miRNAs were identified in tail fat tissue between Hu sheep and Tibetan sheep. Target genes of DE miRNAs were annotated by GO and KEGG. Some lipid metabolism terms and pathways were enriched including lipid metabolic process, response to lipid, cellular lipid catabolic process, lipid catabolic process, cellular lipid metabolic process, inositol lipid-mediated signaling, calcium channel activity, PI3K-Akt signaling pathway, MAPK signaling pathway, ECM-receptor interaction, AMPK signaling pathway, Wnt signal pathway and TGF-beta signaling pathway. Meanwhile, we verified the targeted relationship between miR-379-5p and *HOXC9*. MiR-379-5p was highly expressed in Tibetan sheep. *HOXC9* was highly expressed in adipose tissue of Hu sheep. This study could provide a meaningful theoretical basis for studying the molecular mechanisms of tail fat adipogenesis.

## Supporting information

S1 Table Primers for clone and RT-qPCR of 10 random selected differentially expressed miRNAs. (XLSX)

S2 Table The length of miRNA in each library. (XLSX)

S3 Table The identification of miRNA in each library. (XLSX)

S4 Table The identification of miRNA in two comparisons. (XLSX)

S5 Table The differently expression miRNA. (XLSX)

S6 Table The target relationship of DE miRNAs. (XLSX)

S7 Table The result of GO analysis. (XLSX)

S8 Table The result of KEGG. (XLSX)

S9 Table The sequencing result of psiCHECK2-*HOXC9*-3′UTR-WT. (DOCX)

S10 Table The sequencing result of psiCHECK2-*HOXC9*-3′UTR-MT. (DOCX)

S1 Fig Monoclonals identification by PCR. The positive clones identified by PCR are marked with a red frame.
(TIFF)

S1 Raw image (TIF)
