# Supplementary material for: Transcriptome reveals key microRNAs involved in fat deposition between different tail sheep breeds
Source: PLoS One. 2022 Mar 1;17(3):e0264804. doi: 10.1371/journal.pone.0264804 (PMC8887763; doi:10.1371/journal.pone.0264804)
Supplement: S10 Table — (DOCX) [file pone.0264804.s010.docx]

CATCCAGGCAGTATGTATGAAATAAGATATTTTATTGCGGCCAGCGGCCGCTTGGGTTTCTGGTTTTTTAAATGACTTCGATATATTAACACAGAGCTGTTCTACCATAACAAACAGAAGCACGTTACAGGACCGACTCTGAGGATGAGAGGAGACTGCTATGTACACTCCAGAGGCGCCGGCCTGTGCCGCTGGACCCAGCCTGCGGCATGGTCCCCGGCCCTCGCCTTTTAGCTCCTGGGCTGGGAAGCATACTTGTGTATGTTGTGTGTGTGCGCGTGTGTGTGTTTGTACAAAATCTGCACACAGAGCACCCCAGGCCTGGGGAACGATGGCCCGGACCAAATACAATACATGAAAAAAAATAGAAATATAGCGATTCAAGTACTGGCTTCTCTGAAGAAAGGAGAAAAAAAAAATCACATTTGTGTGTGTCATTTATTTCAGTTGCGCTGGGAGAAGAGAACGCGGTTTCTCTCCCCGCCTCCTCCTCCTCCTCGCTGAAGCTCCCTAACTCTAAAACACCAATTTCTCAACACTGAACCCTCCCAAATAGCAAGAGTTTTCCTTTTCCCCTTCCCTTTTTTTTTTTTTTTTTCCTTTTTAAACCGATTGGCTTTTCTCTATCTTGCTCTTTCCTTTTTTCTTTCAGTGCTGTCTCCCGCCTGGGCTGGGGTATTTTGTGGGTTTTTTGTTTTGTTTTGTTTTTCCCTTGGCTGTGCGAGGCAGCAGGCTGGGGCAGGGTCTCGAGCGATCGCCTAGAATTACTGCTCGTTCTTCAGCACGCGCTCCACGAAGCTCTTGATGTACTTACCCATTTCATCTGGAGCGTCCTCCTGGCTGAAGTGGAGGCCCTTCACCTTCACGAACTCGGTGTTAGGGAACTTCTTAGCTCCCTCGACATAGCGTTGGAAAGACCCAGGGTCGGACTCGATGACATCTAGCAGATCGTCGCTGCCGAGTAGGCGTTGTAGTGCGACATCTGACGACGTCGGCTGCCTCCATAC
