# Supplementary figures and images for: Transcriptome reveals key microRNAs involved in fat deposition between different tail sheep breeds
Source: PLoS One. 2022 Mar 1;17(3):e0264804. doi: 10.1371/journal.pone.0264804 (PMC8887763; doi:10.1371/journal.pone.0264804)

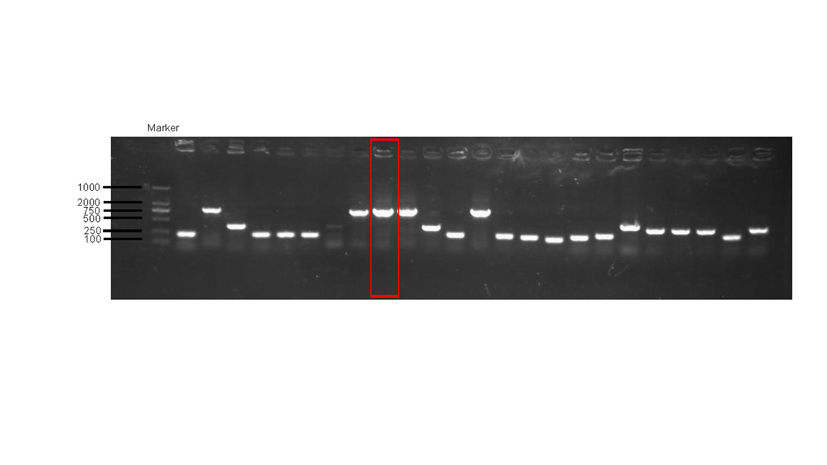

Supplement: S1 Fig — The positive clones identified by PCR are marked with a red frame. (TIFF) [file pone.0264804.s011.tiff]

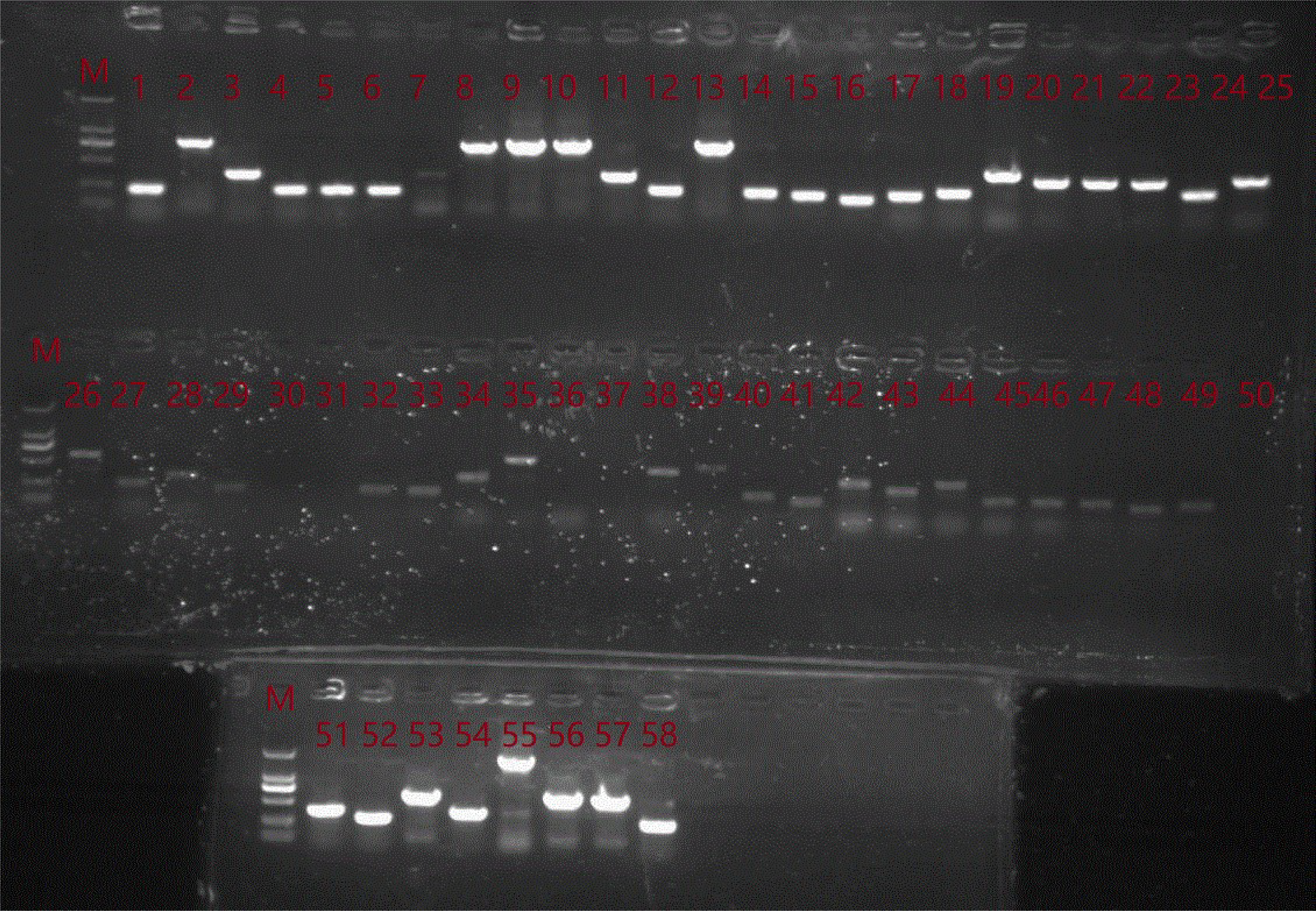

Supplement: S1 Raw image — (TIF) [file pone.0264804.s012.tif]
